# Bespoke contrast-matched diblock copolymer nanoparticles enable the rational design of highly transparent Pickering double emulsions†
†Electronic supplementary information (ESI) available: GPC chromatograms, additional transmission electron micrographs, digital photographs, visible absorption spectra and laser diffraction data, further optical and fluorescence micrographs. See DOI: 10.1039/c6nr03856e



**DOI:** 10.1039/c6nr03856e

**Published:** 2016-07-06

**Authors:** Matthew J. Rymaruk, Kate L. Thompson, Matthew J. Derry, Nicholas J. Warren, Liam P. D. Ratcliffe, Clive N. Williams, Steven L. Brown, Steven P. Armes

**Affiliations:** a Dainton Building , Department of Chemistry , The University of Sheffield , Brook Hill , Sheffield , S3 7HF , Yorkshire , UK . Email: s.p.armes@sheffield.ac.uk ; Email: mjrymaruk1@sheffield.ac.uk; b Scott Bader Company Ltd , Wollaston, Wellingborough , NN29 7RL , Northants , UK

## Abstract

We report the preparation of highly transparent oil-in-water Pickering emulsions using contrast-matched organic nanoparticles. This is achieved *via* addition of judicious amounts of either sucrose or glycerol to an aqueous dispersion of poly(glycerol monomethacrylate)_56_–poly(2,2,2-trifluoroethyl methacrylate)_500_ [PGMA–PTFEMA] diblock copolymer nanoparticles prior to high shear homogenization with an equal volume of *n*-dodecane. The resulting Pickering emulsions comprise polydisperse *n*-dodecane droplets of 20–100 μm diameter and exhibit up to 96% transmittance across the visible spectrum. In contrast, control experiments using non-contrast-matched poly(glycerol monomethacrylate)_56_–poly(benzyl methacrylate)_300_ [PGMA_56_–PBzMA_300_] diblock copolymer nanoparticles as a Pickering emulsifier only produced conventional highly turbid emulsions. Thus contrast-matching of the two immiscible phases is a necessary but not sufficient condition for the preparation of highly transparent Pickering emulsions: it is essential to use isorefractive nanoparticles in order to minimize light scattering. Furthermore, highly transparent oil-in-water-in-oil Pickering double emulsions can be obtained by homogenizing the contrast-matched oil-in-water Pickering emulsion prepared using the PGMA_56_–PTFEMA_500_ nanoparticles with a contrast-matched dispersion of hydrophobic poly(lauryl methacrylate)_39_–poly(2,2,2-trifluoroethyl methacrylate)_800_ [PLMA_39_–PTFEMA_800_] diblock copolymer nanoparticles in *n*-dodecane. Finally, we show that an isorefractive oil-in-water Pickering emulsion enables fluorescence spectroscopy to be used to monitor the transport of water-insoluble small molecules (pyrene and benzophenone) between *n*-dodecane droplets. Such transport is significantly less efficient than that observed for the equivalent isorefractive surfactant-stabilized emulsion. Conventional turbid emulsions do not enable such a comparison to be made because the intense light scattering leads to substantial spectral attenuation.

## Abstract 2

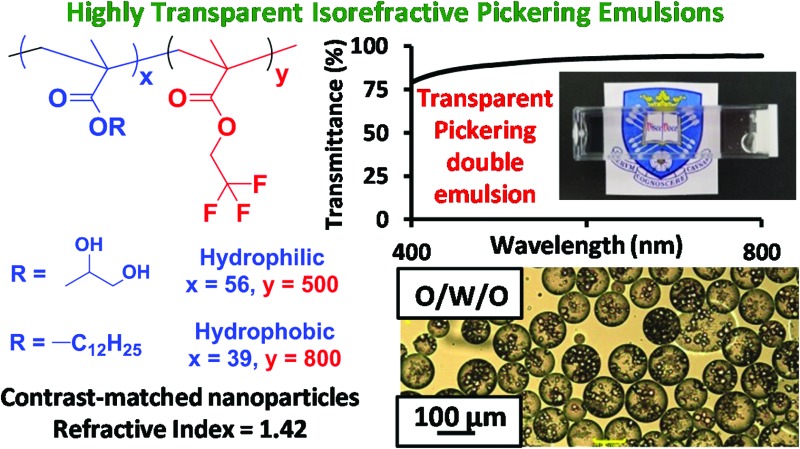
Contrast-matched diblock copolymer nanoparticles facilitate the production of highly transparent Pickering emulsions and Pickering double emulsions.

## Introduction

Ramsden^1^ and Pickering^2^ demonstrated over a century ago that colloidal particles can stabilize emulsions. After many decades of little or no activity, there has been a resurgence of interest in Pickering emulsions over the last 17 years or so.^3^ Many types of particles have now been evaluated in this context, including inorganic materials such as silica,^4–6^ iron oxide,^7^ calcium carbonate,^8^ barium sulfate,^9^ titanium dioxide^10^ or clays^11–13^ and organic materials such as copolymer latexes,^14–26^ cellulosic particles,^27–30^ carbon black,^31^ epoxy resins^32^ and nanocomposite particles.^33^ The driving force for emulsion stability is particle adsorption at the oil/water interface, since this reduces the surface area (and therefore the interfacial energy) of the droplet phase.^34^ The particle contact angle, *θ*, is related to the surface wettability and usually dictates the emulsion type: hydrophilic particles (*θ* < 90°) normally produce oil-in-water emulsions, whereas hydrophobic particles (*θ* > 90°) favor the formation of water-in-oil emulsions.^35–40^ Compared to conventional surfactant-stabilized emulsions, Pickering emulsions offer enhanced long-term stability, reduced foaming and more reproducible formulations.^34^

According to Snell's law, no refraction occurs when light travels between two media with the same refractive index.^41^ This scenario applies to emulsions when the continuous phase and the droplet phase have equal refractive indices and results in transparency.^41^ For surfactant-stabilized emulsions, the emulsifier is too small to cause light scattering (or turbidity). Thus transparent surfactant-stabilized emulsions have been reported for various applications.^41–43^ However, the design of refractive index-matched Pickering emulsions is much more technically challenging. In general, the particles are likely to scatter light, particularly if they are adsorbed at the oil/water interface as aggregates, rather than as individual particles.^44,45^ Thus in this case the droplet phase, continuous phase and the Pickering emulsifier must be contrast-matched for high transparency.

Recently, Binks and co-workers reported the production of translucent non-aqueous Pickering emulsions. This formulation comprised paraffin liquid droplets stabilized by silica nanoparticles, dispersed in a poly(ethylene glycol)_300_ continuous phase.^46^ The relatively small refractive index difference between the two immiscible liquids (1.475 and 1.464, respectively) gave rise to Pickering emulsions of relatively low turbidity. However, the non-contrast matched silica nanoparticles scattered light sufficiently strongly to limit the transparency of this emulsion. Similarly, Thompson and co-workers reported the preparation of a near-isorefractive non-aqueous Pickering emulsion.^47^ This formulation comprised *n*-tetradecane, ethylene glycol and poly(lauryl methacrylate)_16_–poly(benzyl methacrylate)_37_ (PLMA_16_–PBzMA_37_) diblock copolymer worms^48^ as the Pickering emulsifier. However, *n*-tetradecane is relatively expensive, ethylene glycol has significant toxicity and the worms were not contrast-matched, which limited the transmittance to around 70–80% depending on the precise wavelength of visible light. Thus, although of some academic interest, this particular formulation appears to have little or no commercial potential.

As far as we are aware, highly transparent Pickering emulsions have not yet been reported, despite the substantial level of interest in this field. In the present work, we report the preparation of isorefractive oil-in-water (o/w) emulsions and oil-in-water-in-oil (o/w/o) double emulsions using contrast-matched Pickering emulsifiers. This was achieved by designing two new types of sterically-stabilized diblock copolymer nanoparticles each comprising a poly(2,2,2-trifluoroethyl methacrylate) (PTFEMA) core-forming block combined with either (i) a hydrophilic poly(glycerol monomethacrylate) (PGMA) stabilizer block or (ii) a hydrophobic PLMA stabilizer block. The PTFEMA block was chosen for its relatively low refractive index of 1.42;^49^ this almost precisely matches that of *n*-dodecane, which was the model oil used in this study.^50^ The PGMA stabilizer was selected for its exceptional tolerance towards high concentrations of sucrose or glycerol, which were judiciously added to an aqueous dispersion of PGMA–PTFEMA nanoparticles to raise the refractive index of this phase in order to achieve a near-perfect contrast match. The PLMA stabilizer was selected to ensure good colloidal stability for the PLMA–PTFEMA nanoparticles, which were prepared directly in *n*-dodecane.^51^

## Results and discussion

**Fig. 1:**
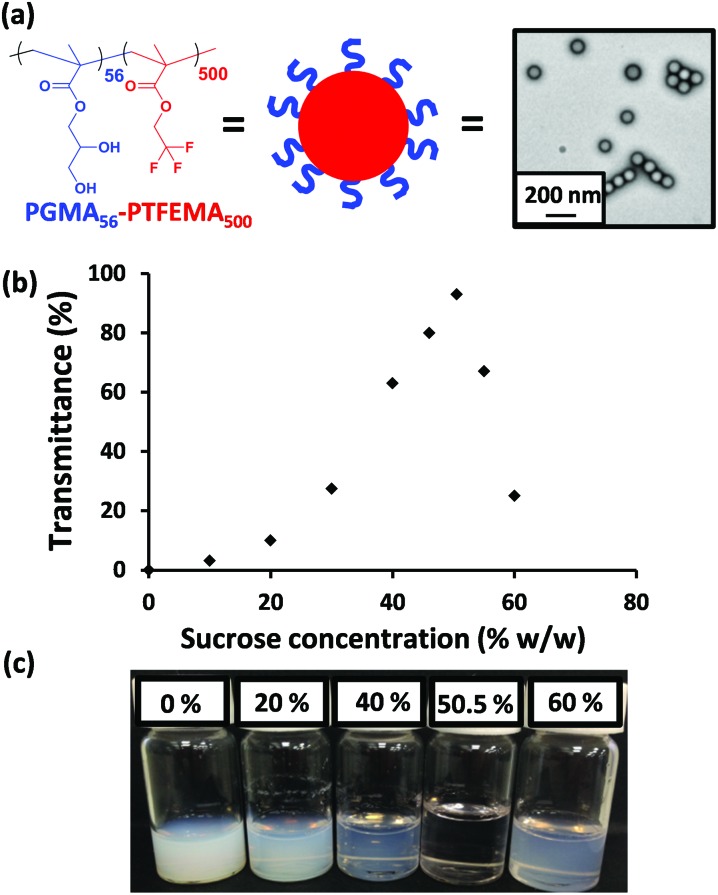
(a) Chemical structure, schematic cartoon and a representative transmission electron microscopy image of the PGMA_56_–PTFEMA_500_ diblock copolymer nanoparticles used in this work. (b) Transmittance data obtained at 400 nm for a 2.0 % w/w dispersion of PGMA_56_–PTFEMA_500_ nanoparticles as a function of sucrose concentration. (c) Corresponding digital images for selected aqueous dispersions in the presence of various sucrose concentrations.

A poly(glycerol monomethacrylate) macro-chain transfer agent (PGMA_56_ macro-CTA) was prepared *via* RAFT solution polymerization in ethanol at 70 °C using 2-cyano-2-propyl dithiobenzoate (CPDB). This near-monodisperse precursor (mean degree of polymerization, DP = 56; *M*_w_/*M*_n_ = 1.20) was then chain-extended *via* the RAFT aqueous emulsion polymerization of TFEMA at 15 % w/w solids (target DP = 500). ^1^H and ^19^F NMR spectroscopy studies confirmed a mean diblock composition of PGMA_56_–PTFEMA_500_ (see Fig. 1a), while gel permeation chromatography (GPC) analysis indicated a relatively low final *M*_w_/*M*_n_ of 1.25. Transmission electron microscopy (TEM) analysis confirmed a well-defined spherical morphology for these diblock copolymer nanoparticles (see Fig. S1†) and dynamic light scattering (DLS) studies indicated a *z*-average diameter of 101 nm.

The as-synthesized 15 % w/w aqueous dispersion of PGMA_56_–PTFEMA_500_ nanoparticles was highly turbid, as expected given the relatively large refractive index difference between the major PTFEMA component (1.42) and pure water (1.33). To produce a highly transparent dispersion, sucrose was gradually added to a 2.0 % w/w aqueous dispersion of PGMA_56_–PTFEMA_500_ nanoparticles in order to achieve isorefractivity (see Fig. 1b). The ensuing reduction in turbidity could be conveniently monitored by visible absorption spectroscopy. As the aqueous sucrose concentration was increased from zero up to approximately 50 % w/w, the transmittance of the aqueous dispersion at 400 nm increased dramatically from approximately 0% up to 98%. However, higher sucrose concentrations led to a reduction in transmission. Thus, 50.5 % w/w sucrose corresponds to a contrast-matched dispersion with maximum transmittance. This indicates that the refractive index of these sterically-stabilized nanoparticles is approximately 1.42 (*i.e.* the same as that of a 50.5 % w/w aqueous sucrose solution, see Fig. S2a†).^52^ Hence this parameter is primarily governed by the refractive index of the core-forming PTFEMA block and the influence of the highly solvated PGMA stabilizer chains is negligible.

Similar experiments using glycerol instead of sucrose confirmed that a similarly transparent dispersion could be obtained when the aqueous continuous phase contained 65 % w/w of the alcoholic co-solvent (see Fig. S3†). This observation is consistent with the literature: the refractive index of such a glycerol-rich aqueous solution is known to be approximately 1.42 (Fig. S2b†).^53^ It is perhaps noteworthy that the latter formulation may be of potential interest for transparent cosmetics formulations, since glycerol is cheap, non-toxic and a well-known humectant.^54^

**Fig. 2:**
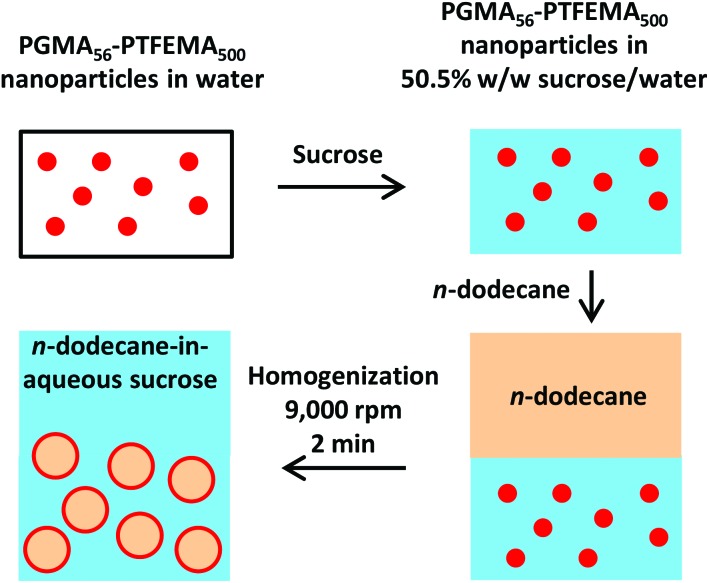
Schematic preparation of *n*-dodecane-in-50.5 % w/w aqueous sucrose Pickering emulsions with 1.2–3.5 % w/w spherical nanoparticles dispersed in the continuous phase.

**Fig. 3:**
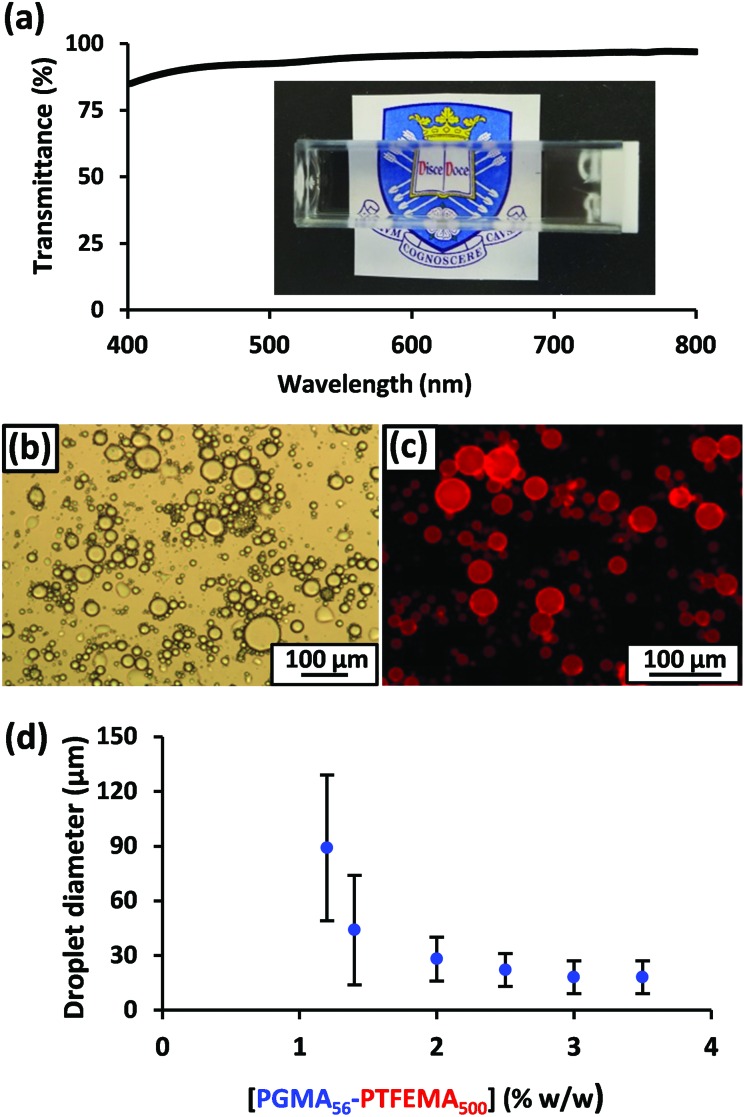
(a) Digital photograph of *n*-dodecane-in-50.5 % w/w aqueous sucrose Pickering emulsion prepared using 1.2 % w/w PGMA_56_–PTFEMA_500_ spherical nanoparticles and the corresponding transmittance data. (b) Optical micrograph obtained for the same emulsion after dilution using pure water. (c) Fluorescence micrograph of this emulsion with the hydrophobic dye, Nile Red, dissolved in the *n*-dodecane droplet phase. (d) Variation in volume-average droplet diameter (as determined by laser diffraction) *vs*. PGMA_56_–PTFEMA_500_ copolymer concentration. The error bars represent the standard deviation of each mean volume-average diameter.

For emulsification experiments, a series of isorefractive aqueous sucrose dispersions of PGMA_56_–PTFEMA_500_ nanoparticles were prepared at copolymer concentrations ranging from 1.2% to 3.5 % w/w. Each of these dispersions were then homogenized in turn with an equal volume of *n*-dodecane at 9000 rpm for 2 min to produce contrast-matched Pickering emulsions (see Fig. 2). A digital photograph (Fig. 3a) of an *n*-dodecane-in-50.5% aqueous sucrose Pickering emulsion prepared using 1.20 % w/w PGMA_56_–TFEMA_500_ nanoparticles serves to illustrate the remarkably high transparency that can be achieved. Visible absorption spectroscopy studies indicated an average transmittance of 96% at 20 °C (ref. 55) (see Fig. 3a). Optical microscopy was used to confirm that stable Pickering emulsions had been formed. Initially, the *n*-dodecane droplets could not be observed, because of the almost perfect isorefractivity. This problem was overcome by diluting each Pickering emulsion with pure water (rather than ∼50% aqueous sucrose solution) prior to visual inspection. This protocol resulted in sufficient contrast to visualize the oil droplets (see Fig. 3b). The ease of dilution of the Pickering emulsions using pure water indicated that the aqueous sucrose solution was indeed the continuous phase, as expected. This was confirmed by conductivity studies and is consistent with the observation that the less dense *n*-dodecane droplets (density of *n*-dodecane = 0.75 g cm^–3^)^50^ gradually creamed on standing at 20 °C. Laser diffraction studies performed on dilute emulsions indicated that large polydisperse droplets with a mean diameter of 89 ± 40 μm were produced when using 1.20 % w/w PGMA_56_–PTFEMA_500_ nanoparticles. Using a higher nanoparticle concentration of 3.5 % w/w leads to the formation of smaller droplets of 20 ± 9 μm diameter. These observations were corroborated by dissolving Nile Red in *n*-dodecane prior to homogenization: this hydrophobic water-insoluble dye enables the resulting Pickering emulsions to be imaged *via* fluorescence microscopy (Fig. 3c). The pronounced upturn in droplet diameter on lowering the nanoparticle concentration (Fig. 3d) is characteristic of a Pickering emulsifier and has been widely reported in the literature.^56–62^ Similar experiments conducted using 65% glycerol instead of ∼50% aqueous sucrose also produced highly-transparent Pickering emulsions with a mean droplet diameter of 85 ± 45 μm and an average transmittance of 95% (see Fig. S4†). To investigate the importance of contrast-matching the nanoparticles as well as the two immiscible liquids, the same PGMA_56_ macro-CTA was also used to conduct the RAFT aqueous emulsion polymerization of benzyl methacrylate, as described previously by Cunningham and co-workers.^63^ PBzMA was selected for the core-forming block as its refractive index of 1.57 ^64^ is significantly higher than that of PTFEMA, *n*-dodecane and ∼50% aqueous sucrose (each approximately 1.42). ^1^H NMR spectroscopy analysis indicated more than 99% BzMA conversion, while DLS studies indicated a *z*-average diameter of 102 nm for the resulting PGMA_56_–PBzMA_300_ nanoparticles, which is comparable to that of the PGMA_56_–TFEMA_500_ nanoparticles. Because the former nanoparticles are not contrast-matched to the two isorefractive immiscible liquids, this new formulation serves as a useful control experiment. Sucrose was added to a 10 % w/w aqueous dispersion of PGMA_56_–PBzMA_300_ nanoparticles to obtain a final sucrose concentration of 50.5 % w/w. This dispersion was then diluted using 50.5% aqueous sucrose to produce a final copolymer concentration of 1.20 % w/w, followed by homogenization with an equal volume of *n*-dodecane at 9000 rpm for 2 min. Optical microscopy studies confirmed that a stable Pickering emulsion was formed, with laser diffraction analysis indicating a mean droplet diameter of 40 ± 18 μm (see Fig. S5a†). However, in this case visible absorption spectroscopy studies of the Pickering emulsion indicated a mean transmittance of approximately 0% across the entire wavelength range, which is characteristic of a highly turbid emulsion (see Fig. S5a†). Similar experiments using 65 % w/w aqueous glycerol instead of sucrose also produced conventional turbid emulsions with an average transmittance of ∼0% across the visible spectrum (see Fig. S5b†). Hence these control experiments confirm the importance of contrast-matching the nanoparticle emulsifier in addition to using isorefractive immiscible liquids if highly transparent Pickering emulsions are desired.

Having rationally designed transparent oil-in-water Pickering emulsions, highly transparent Pickering double emulsions were targeted. Various examples of conventional (*i.e.* turbid) Pickering double emulsions have been reported^65,66^ and potential applications for the encapsulation of various actives have been suggested.^67–69^According to the literature,^34,70,71^ such formulations require the design and use of hydrophobic nanoparticles to supplement the hydrophilic PGMA_56_–PTFEMA_500_ nanoparticles. This is because the former nanoparticles are required to stabilize water-in-oil emulsions,^35^ whereas the latter invariably favor the formation of oil-in-water emulsions (*vide supra*). Thus a poly(lauryl methacrylate)_39_ (PLMA)_39_ macro-CTA was used to synthesize new hydrophobic PLMA_39_–PTFEMA_800_ nanoparticles *via* RAFT dispersion polymerization of TFEMA at 10 % w/w in *n*-dodecane, using a PISA formulation similar to that reported by Fielding and co-workers.^51^ Both ^19^F and ^1^H NMR spectroscopy indicated >99% TFEMA conversion. DLS studies indicated near-monodisperse nanoparticles with a *z*-average diameter of 93 nm, while TEM studies confirmed a well-defined spherical morphology. This PLMA_39_–PTFEMA_800_ dispersion was highly transparent even at 10 % w/w solids, suggesting that the refractive index of the nanoparticles is essentially the same as that of *n*-dodecane (1.42).

**Fig. 4:**
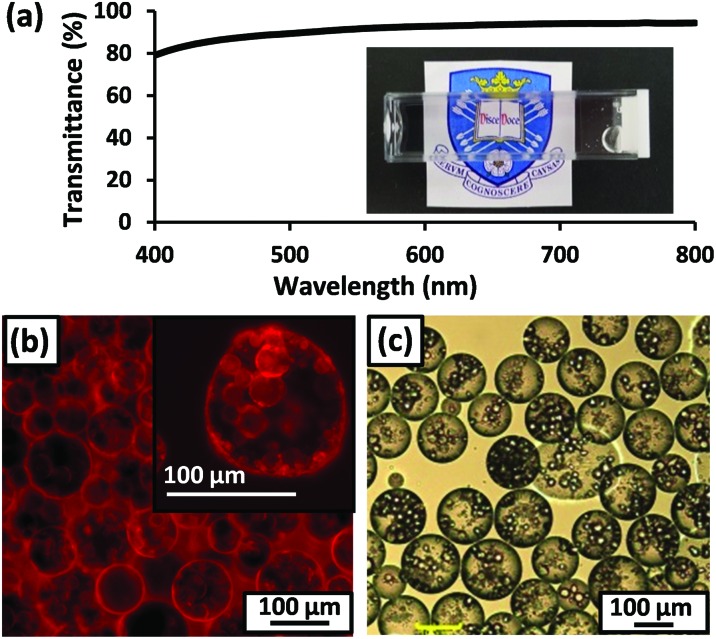
(a) Digital photograph of *n*-dodecane-in-50.5% aqueous sucrose-in-*n*-dodecane Pickering double emulsion with the corresponding transmittance data. (b) Fluorescence micrograph obtained for such an emulsion prepared with Nile Red dissolved in both the internal and external *n*-dodecane phases. (c) Optical micrograph obtained for the same emulsion prepared in the absence of any sucrose, *i.e.* with pure water, in order to provide contrast.

Pickering double emulsions were then prepared as follows. First, the precursor oil-in-water emulsion was prepared using 2.0 % w/w hydrophilic PGMA_56_–PTFEMA_500_ nanoparticles dispersed in a 50.5 % w/w aqueous sucrose solution, an *n*-dodecane volume fraction of 0.50 and a shear rate of 24 000 rpm. These conditions were selected to produce the smallest possible droplets (23 ± 12 μm diameter as judged by laser diffraction) in order to maximize the probability of their encapsulation within the aqueous droplets formed during the second-stage emulsification. This precursor emulsion was then homogenized with an equal volume of *n*-dodecane containing 2.0 % w/w hydrophobic PLMA_39_–PTFEMA_800_ nanoparticles at a shear rate of 7000 rpm. Laser diffraction analysis of the resulting Pickering double emulsion indicated a mean aqueous droplet diameter of 120 ± 68 μm. A digital photograph of the final Pickering double emulsion confirmed its relatively high transparency, with visible absorption spectroscopy studies indicating a mean transmittance of 89% (Fig. 4a). Dissolving Nile Red in both the initial batch of *n*-dodecane (*i.e.* that used to generate the oil-in-water precursor emulsion), and also the second batch of *n*-dodecane enabled imaging *via* fluorescence microscopy (Fig. 4B). These studies indicated successful formation of a Pickering double emulsion comprising relatively small *n*-dodecane droplets within larger droplets of ∼50 % w/w aqueous sucrose, with *n*-dodecane forming the continuous phase. These observations were consistent with sedimentation of the relatively dense aqueous droplet phase on standing. Although prone to sedimentation on standing, laser diffraction studies confirmed that these Pickering double emulsions nevertheless remained stable with respect to coalescence after storage for up to 3 days at 20 °C. Image analysis of fluorescence micrographs recorded for these double emulsions using ImageJ software indicated that the inner *n*-dodecane droplets had a mean diameter of approximately 21 μm, which is comparable to that observed for the precursor single emulsion (23 ± 12 μm as judged by laser diffraction). This suggests that no significant change in droplet diameter occurred during the second-stage homogenization. Finally, the above double emulsification protocol was repeated using pure water (*i.e.* in the absence of any sucrose) to provide sufficient contrast for optical microscopy studies, which confirmed that the aqueous droplets contained much smaller *n*-dodecane droplets within them (see Fig. 4c).

**Scheme 1:**
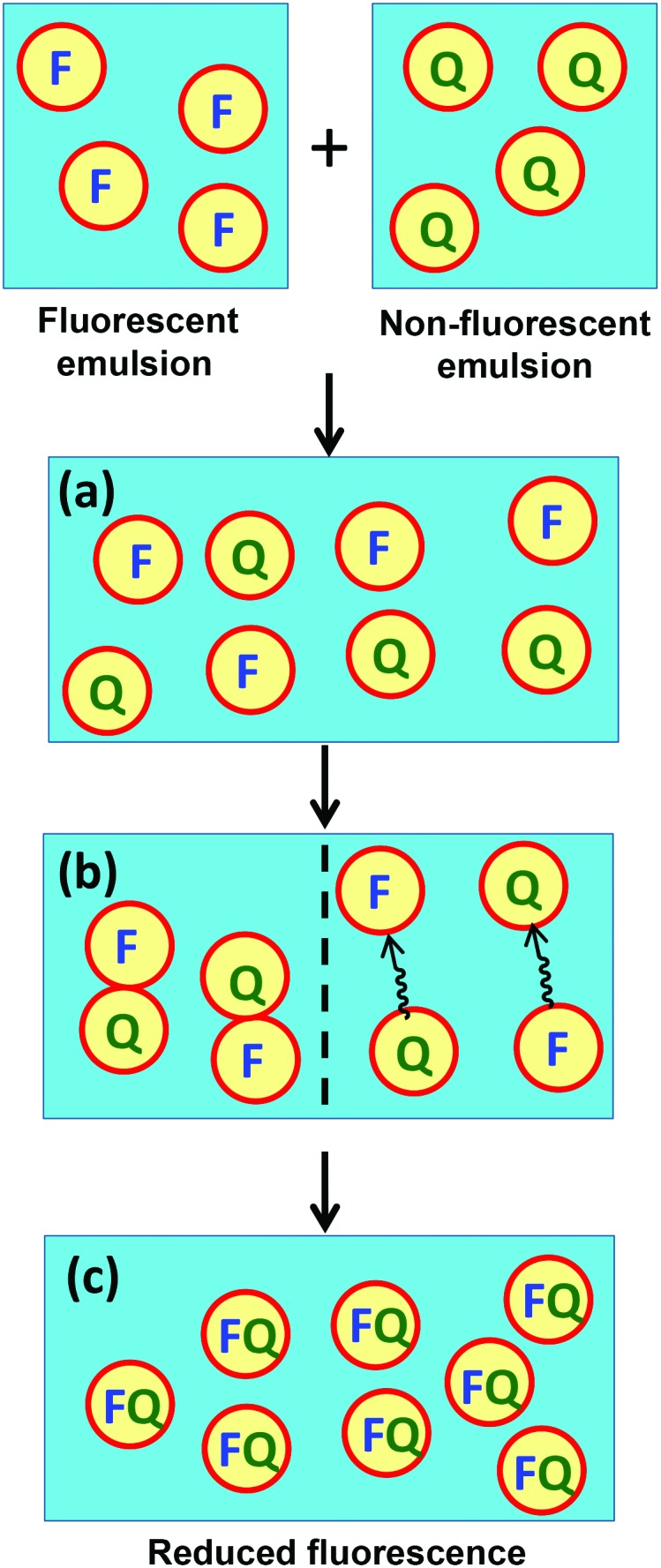
Schematic representation of the mixing of two *n*-dodecane-in-aqueous sucrose emulsions. In each case, one emulsion contains a fluorophore (F; 20 μM pyrene) and the other contains a quencher (Q; 50 mM benzophenone). (a) On initial mixing of the two emulsions, the fluorophore and quencher droplets remain distinct species. (b) After a certain time period, mass transport of the quencher (and/or fluorophore) occurs between neighbouring droplets. Two possible mass transport mechanisms are shown: inter-droplet collisions (left) and diffusion through the aqueous solution *via* Ostwald ripening (right). (c) Eventually, the pyrene fluorescence is effectively quenched by the presence of benzophenone.

**Fig. 5:**
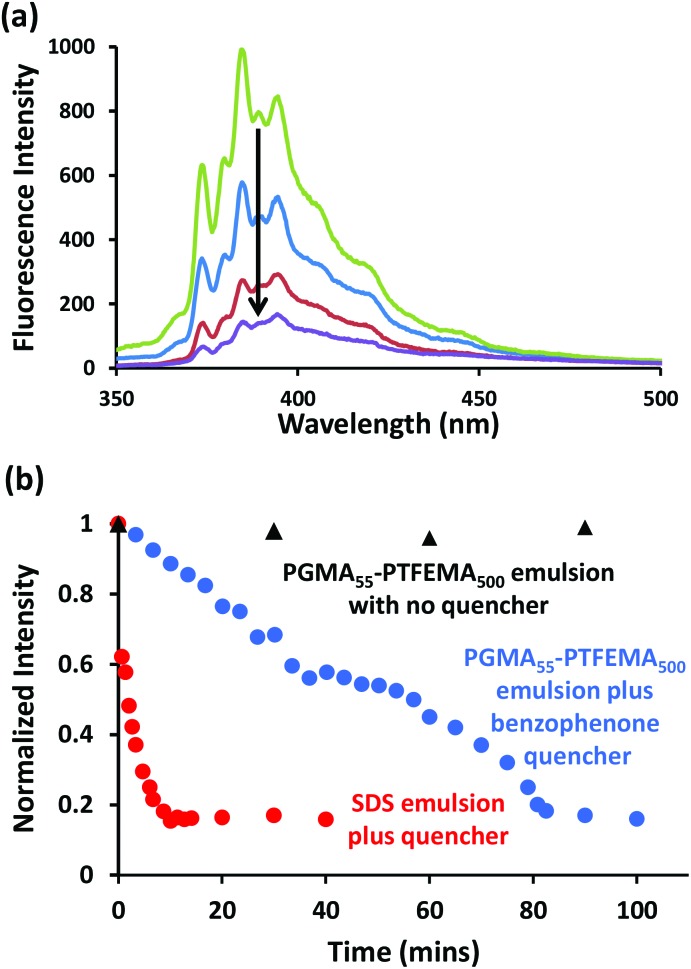
(a) Pyrene emission spectra recorded at 20 °C after 0 min (green), 30 min (blue), 60 min (red) and 90 min (purple) for a PGMA_55_–PTFEMA_500_ nanoparticle-stabilized *n*-dodecane-in-water Pickering emulsion (*n*-dodecane volume fraction = 0.50; isorefractive aqueous phase contained 50.5% sucrose) containing 20 μM pyrene mixed with an equal volume of the same Pickering emulsion containing 50 mM benzophenone as a quencher. (b) Normalized fluorescence intensity at 384 nm recorded as a function of time for the PGMA_55_–PTFEMA_500_-stabilized and SDS-stabilized emulsions in the presence of benzophenone quencher. The control experiment conducted in the absence of benzophenone is also shown. The SDS concentration was 0.004 % w/w and the PGMA_55_–PTFEMA_500_ nanoparticle concentration was 2.0 % w/w respectively, corresponding to a mean oil droplet diameter of approximately 40 μm in each case. The excitation wavelength was 319 nm, the scan speed was 240 nm min^–1^, the PMT voltage was set at 950 V, the excitation slit width was 5 nm and the emission slit width was 2.5 nm.

The transparency of these contrast-matched Pickering emulsions offers an unprecedented opportunity to examine the extent of mass transport between droplets using fluorescence spectroscopy. Thus two isorefractive oil-in-water Pickering emulsions were prepared under identical conditions (9000 rpm for 2 min using 2.0 % w/w PGMA_55_–PTFEMA_500_ nanoparticles in 50.5 % w/w aqueous sucrose and 50 vol% *n*-dodecane) to afford *n*-dodecane droplets of approximately 39 μm diameter, see Fig. S5a.† One emulsion contained 20 μM pyrene as a fluorophore while the second emulsion contained 50 mM benzophenone as a fluorescence quencher, see Scheme 1.^72^ These two reagents were selected because of their relatively low water solubilities, which were expected to minimize mass transport *via* diffusion through the aqueous sucrose continuous phase. [In this context, it is perhaps worth noting that pyrene is more than two orders of magnitude less soluble in water than benzophenone, so if Ostwald ripening were to occur for this system it is more likely to involve the quencher than the fluorophore]. On mixing these two Pickering emulsions at 20 °C, the pyrene spectrum gradually became attenuated over 90 min, see Fig. 5a. In contrast, significantly faster quenching was observed for pyrene dissolved in a surfactant-stabilized emulsion (see Fig. 5b). The latter emulsion was prepared using 0.004 M sodium dodecylsulfate (SDS) and had a mean volume-average diameter of 42 μm (see Fig. S6a†), hence any surface area differences should be negligible. Two control experiments were also performed as part of this fluorescence spectroscopy study. First, a pyrene-loaded Pickering emulsion was added to a second emulsion containing no benzophenone. In this case essentially no reduction in pyrene fluorescence was observed (see Fig. 5b), which demonstrates that the attenuation in fluorescence intensity observed in the presence of benzophenone is indeed caused by this well-known pyrene quencher.^72^ Second, a conventional highly turbid Pickering emulsion was prepared using PGMA_55_–PBzMA_300_ nanoparticles dispersed in water to stabilize *n*-dodecane droplets containing 20 μM pyrene. As expected, the intense light scattering for this system leads to almost complete attenuation of the pyrene spectrum (see Fig. S6b†), which prevents the mass transport of water-insoluble species from being conveniently monitored.

At first sight, the observations summarized in Fig. 5 suggest that Pickering emulsions provide a more effective barrier towards inter-droplet mass transport than SDS-stabilized emulsions. However, the presence of sucrose may in principle increase the solubility of either pyrene or benzophenone in the aqueous continuous phase. Indeed, further fluorescence studies (see Fig. S7a†) indicated that pyrene is approximately an order of magnitude more soluble in 50.5% aqueous sucrose than in pure water, whereas UV spectroscopy studies (Fig. S7b†) confirmed that the solubility of benzophenone remained almost unchanged in the presence of sucrose. In addition, 0.014 M SDS leads to additional solubilization of either the fluorophore or the quencher in the aqueous continuous phase, possibly in the form of micelles (see Fig. S7a†). However, Taylor reported^73^ that SDS micelles had an unexpectedly weak effect on the Ostwald ripening of 100–150 nm diameter SDS-stabilized *n*-decane-in-water emulsions for oil volume fractions up to 0.30. Moreover, Ostwald ripening is not considered to be important for the much larger multimicron-sized emulsions of the type prepared in the present study.^73,74^ In summary, regardless of the precise transport mechanism, the fluorescence studies reported in Fig. 5 suggest that exchange of water-insoluble species between *n*-dodecane droplets is significantly slower for Pickering emulsions than for SDS-stabilized emulsions of comparable size.

## Conclusions

Highly transparent oil-in-water Pickering emulsions can be prepared by the judicious addition of sucrose or glycerol to an aqueous dispersion of relatively low refractive index PGMA_56_–PTFEMA_500_ nanoparticles, followed by high shear homogenization with an isorefractive oil such as *n*-dodecane. The resulting contrast-matched emulsions can exhibit up to 96% transmittance and are stable for months on standing at 20 °C. Control experiments conducted with relatively high refractive index nanoparticles (*e.g.* PGMA_56_–PBzMA_300_) confirm that contrast-matching the aqueous phase with the oil phase is a necessary but not sufficient criterion for a highly transparent Pickering emulsion. This is because if the nanoparticles are not also contrast-matched to the two liquid phases, they scatter light sufficiently strongly to generate substantial turbidity.

Moreover, it is shown that such isorefractive oil-in-water Pickering emulsions enable fluorescence spectroscopy to be used to monitor transport of water-insoluble small molecules (pyrene and benzophenone) between *n*-dodecane droplets, most likely *via* inter-droplet collisions, but possibly *via* diffusion across the aqueous continuous phase. Such transport is significantly slower than that observed for the equivalent isorefractive surfactant-stabilized emulsion. Conventional turbid emulsions do not enable such a comparison to be made because the intense light scattering leads to substantial spectral attenuation. Complementary highly transparent water-in-oil emulsions can be prepared using contrast-matched hydrophobic PLMA_39_–PTFEMA_800_ nanoparticles prepared in *n*-dodecane. Moreover, the judicious combination of these two types of hydrophilic and hydrophobic nanoparticle emulsifiers enables the production of an oil-in-water-in-oil Pickering double emulsion that exhibits a mean transmittance of almost 90% across the visible spectrum. Such studies serve to illustrate the remarkable versatility and tremendous potential offered by polymerization-induced self-assembly (PISA) for the rational design of organic nano-objects of tunable size, morphology and surface chemistry as bespoke Pickering emulsifiers with a high degree of dispersion prior to adsorption at the oil/water interface.

## Experimental

### Materials

Glycerol monomethacrylate (GMA, purity 97%) was obtained from GEO speciality chemicals (Hythe, UK) and was used as received. 2,2,2-Trifluoroethylmethacrylate (TFEMA, 99%), lauryl methacrylate (LMA, 96%), *n*-dodecane (>99%), glycerol (>99%), sucrose (>99.5%), Nile red, CD_3_OD, tetrahydrofuran (THF), dimethylformamide (DMF) (CD_3_)_2_CO, lithium bromide (LiBr), CDCl_3_, dimethyl sulfoxide (DMSO), triethylamine, 3,5-di-*tert*-4-butylhydroxytoluene (BHT), toluene, benzyl methacrylate (BzMA, 96%), 4,4′-azobis(4-cyanovaleric acid) (ACVA, >97%), benzophenone (>99%), pyrene (>99%), 2-cyanopropyldithiobenzoate (CPDB, >97%), 2-phenylethanethiol, sodium hydride (60% in mineral oil), diethyl ether, carbon disulfide, iodine, sodium thiosulfate, sodium sulfate, ethyl acetate and *n*-hexane were purchased from Sigma Aldrich (UK). *tert*-Butyl peroxy-2-ethylhexanoate (Trigonox 21S or T21s) initiator was supplied by AkzoNobel (The Netherlands) and sodium dodecylsulfate (SDS) was obtained from BDH laboratory supplies (Poole, UK). Benzyl methacrylate was passed through basic alumina prior to use; all remaining reagents were used as received unless otherwise stated. Deionized water (pH 6.1 at 20 °C) was used for all experiments described herein. All solvents used were of HPLC grade.

### Synthesis of 4-cyano-4-(2-phenylethane sulfanylthiocarbonyl)sulfanylpentanoic acid (PETTC)

2-Phenylethanethiol (21 g, 152 mmol) was added dropwise to a stirred suspension of sodium hydride (60% in oil, 6.3 g, 158 mmol) in diethyl ether (250 mL) at 0 °C. Evolution of hydrogen was observed and the gray suspension turned to a white slurry of sodium phenylethanethiolate over 45 minutes. Carbon disulfide (12.0 g, 158 mmol) was added dropwise and a yellow precipitate of sodium 2-phenylethanetrithiocarbonate formed over 30 minutes, which was collected *via* filtration and used without further purification. To a suspension of sodium 2-phenylethanetrithiocarbonate (23.2 g, 98 mmol) in diethyl ether (150 mL), solid iodine (12.6 g, 50 mmol) was added. The reaction mixture was stirred for 60 minutes at room temperature, and the resulting precipitate of sodium iodide was removed *via* filtration. The brown filtrate was washed with a saturated solution of sodium thiosulfate (2 × 150 mL), dried over sodium sulfate and placed under reduced pressure to leave bis-(2-phenylethane sulfanylthiocarbonyl)disulfide as an orange solid (∼100% yield). A solution of bis-(2-phenylethane sulfanylthiocarbonyl)disulfide (10 g, 23 mmol) and 4,4′-azobis(4-cyanovaleric acid) (9.67 g, 34.5 mmol) in ethyl acetate (250 mL) was purged with nitrogen for 30 minutes at 20 °C before being heated to reflux under a dry nitrogen atmosphere for 18 h. The resulting solution was washed with water (5 × 200 mL), dried over sodium sulfate and placed under reduced pressure to remove the volatiles. The remaining orange residue was recrystallized from ethyl acetate : hexane (4 : 1 v/v) to yield 4-cyano-4-(2-phenylethane sulfanylthiocarbonyl)sulfanylpentanoic acid (PETTC) as a yellow solid (yield 74%): ^1^H NMR (400.13 MHz, CD_2_Cl_2_, 298 K): *δ* 1.91 (3H, CH_3_), 2.41–2.62 (m, 2H, CH_2_), 2.72 (t, 2H, CH_2_), 3.04 (t, 2H, CH_2_), 3.63 (t, 2H, CH_2_), 7.3–7.4 (m, 5H, aromatic). ^13^C NMR (400.13 MHz, CD_2_Cl_2_, 298 K): *δ* 24.4 (CH_3_), 29.6 (CH_2_ *C*H_2_COOH), 30.2 (*C*H2Ph), 33.2 (*C*H2CH2COOH), 40.0 (S*C*H_2_-CH_2_Ph), 45.7 (S*C*CH2), 118.7 (CN), 127.3, 128.9, 129.2, 144.2 (Ph), 177.5 (C=O), 222.2 (C=S).

### Synthesis of poly(glycerol monomethacrylate) macro-chain transfer agent

A poly(glycerol monomethacrylate)_56_ macro-CTA and a poly(glycerol monomethacrylate)_55_ macro-CTA were synthesized *via* RAFT solution polymerization at 40 % w/w in ethanol according to a previously reported protocol.^75^

### Synthesis of poly(lauryl methacrylate) macro-CTA

A typical synthesis of a PLMA_39_ macro-CTA was conducted as follows. A 250 mL round-bottomed flask was charged with lauryl methacrylate (LMA; 18.7 g; 73.5 mmol), 4-cyano-4-(2-phenylethane sulfanylthiocarbonyl)sulfanylpentanoic acid (PETTC; 0.50 g; 1.47 mmol; target degree of polymerization, DP = 50), 2,2′-azobisisobutyronitrile (AIBN; 48.3 mg, 294 μmol; [CDB]/[AIBN] molar ratio = 5.0) and toluene (19.2 g; total solids content = 50 % w/w). The sealed reaction vessel was purged with nitrogen and placed in a pre-heated oil bath at 70 °C for 3.5 h. The resulting PLMA_39_ (LMA conversion = 63%; CTA efficiency = 81%; *M*_n_ = 8200 g mol^–1^, *M*_w_/*M*_n_ = 1.18) was purified by twice precipitating into excess methanol.

### Synthesis of PGMA_56_–PTFEMA_500_ diblock copolymer spheres

A typical RAFT emulsion polymerization of PGMA_56_–PTFEMA_500_ at 15 % w/w was conducted as follows. PGMA_56_ macro-CTA (0.3 g, 0.033 mmol) and ACVA initiator (2.3 mg, 0.0083 mmol) were dissolved in water (15.2 g). The reaction mixture was then sealed in a round-bottomed flask, submerged in an ice bath and purged with nitrogen for 25 minutes. TFEMA monomer was separately purged with nitrogen for 15 minutes before being transferred (2.3 ml, 16.3 mmol) to the reaction mixture. The resulting deoxygenated emulsion was submerged in an oil bath at 70 °C for 8 h (final TFEMA conversion by ^19^F NMR = 98%, *M*_n_ = 72 000 g mol^–1^, *M*_w_ = 89 000 g mol^–1^, *M*_w_/*M*_n_ = 1.25).

### Synthesis of PLMA_39_–PTFEMA_800_ diblock copolymer spheres

A typical RAFT dispersion polymerization of PLMA_39_–PTFEMA_800_ at 10 % w/w was conducted as follows. PLMA_39_ macro-CTA (0.2 g, 0.019 mmol) and T21s initiator (1.0 mg, 0.0048 mmol) were dissolved in *n*-dodecane (25.42 g). The reaction mixture was then sealed in a round-bottomed flask, submerged in an ice bath and purged with nitrogen for 25 minutes. TFEMA monomer was separately purged with nitrogen for 15 minutes before being transferred (2.22 ml, 15.6 mmol) to the reaction mixture. The resulting deoxygenated solution was submerged in an oil bath at 90 °C for 8 h (final TFEMA conversion by ^19^F NMR = 99%, *M*_n_ = 132 000 g mol^–1^, *M*_w_ = 163 000 g mol^–1^, *M*_w_/*M*_n_ = 1.64).

### Synthesis of PGMA_56_–PBzMA_300_ diblock copolymer spheres

PGMA_56_–PBzMA_300_ spherical nanoparticles were prepared *via* RAFT aqueous emulsion polymerization at 10 % w/w according to a previously-reported protocol. Final BzMA conversion by ^1^H NMR = 99%, *M*_n_ = 59 000 g mol^–1^, *M*_w_ = 71 400 g mol^–1^, *M*_w_/*M*_n_ = 1.21).

### Preparation of O/W isorefractive emulsions using glycerol

The as-prepared 15 % w/w PGMA_56_–PTFEMA_500_ aqueous dispersion was diluted with glycerol until a 65 % w/w glycerol/water mixture was reached. The resulting 5.8 % w/w PGMA_56_–PTFEMA_500_ dispersion in 65% aqueous glycerol was then serially diluted with pre-prepared 65 % w/w aqueous glycerol to obtain copolymer concentrations ranging from 1.5 to 4.0 wt%. To prepare the contrast-matched Pickering emulsion, a dilute sphere dispersion (2.0 mL) was homogenized with *n*-dodecane (2.0 mL) for 2.0 minutes using a IKA Ultra-Turrax T-18 homogenizer with a 10 mm dispersing tool operating at 9000 rpm.

### Preparation of O/W isorefractive emulsions using sucrose

Sucrose was added to the as-prepared 15 % w/w PGMA_56_–PTFEMA_500_ aqueous dispersion until a 50.5 % w/w sucrose/water mixture was reached. The resulting 7.4 % w/w PGMA_56_–PTFEMA_500_ dispersion in ∼50% aqueous sucrose was then serially diluted with pre-prepared 50 % w/w aqueous sucrose to obtain copolymer concentrations ranging from 1.2 to 3.5 % w/w. To prepare the contrast-matched Pickering emulsion, a dilute dispersion of PGMA_56_–PTFEMA_500_ nanoparticles (2.0 mL) was homogenized with *n*-dodecane (2.0 mL) for 2.0 minutes using a IKA Ultra-Turrax T-18 homogenizer with a 10 mm dispersing tool operating at 9000 rpm.

### Preparation of O/W/O isorefractive Pickering double emulsion

A single contrast-matched O/W emulsion stabilized by 2.0 % w/w PGMA_56_–PTFEMA_500_ nanoparticles was prepared at 24 000 rpm as above. 2.0 mL of this single O/W emulsion was then homogenized at 20 °C with 2.0 mL of a 2.0 % w/w dispersion of PLMA_39_–PTFEMA_500_ in *n*-dodecane, for 2.0 minutes at 7000 rpm.

### Pyrene quenching experiments

All pyrene emission spectra were recorded from 325 to 700 nm on a PC-controlled Hitachi F-4500 fluorescence spectrophotometer using the following parameters: PMT voltage = 950 V, excitation wavelength = 319 nm, scan rate = 240 nm min^–1^, excitation slit width = 5 nm and emission slit width = 2.5 nm. The pyrene fluorescence intensity was also monitored continuously at 384 nm (excitation wavelength = 319 nm, excitation slit width = 5 nm and an emission slit width = 2.5 nm) during quenching experiments. Pickering emulsions were prepared by dispersing 2.0 % w/w PGMA_55_–PTFEMA_500_ nanoparticles in 50.5 % w/w sucrose and homogenizing with *n*-dodecane at 9000 rpm for 2.0 min at an oil volume fraction of 0.50. The oil droplet phase contained either 20 μM pyrene, 50 mM benzophenone or was pure *n*-dodecane. Quenching experiments were performed by mixing equal volumes of contrast-matched Pickering emulsions containing pyrene and benzophenone and recording the fluorescence emission spectra of the binary emulsion at regular time intervals. A reduction in fluorescence intensity at 384 nm was recorded over time in the presence of benzophenone, which is a well-known quencher for pyrene. In a control experiment, a pyrene-loaded emulsion was mixed with an *n*-dodecane emulsion containing no quencher. This binary emulsion was also monitored over time and essentially no reduction in fluorescence intensity was observed, as expected. Surfactant-stabilized emulsions containing the same concentrations of pyrene and benzophenone were also prepared using 0.004 % w/w SDS in 50.5 % w/w aqueous sucrose.

### Determination of pyrene concentration in 50.5 % w/w aqueous sucrose

20 μM pyrene was dissolved in 4.0 ml *n*-dodecane and hand-shaken with 4.0 ml of either pure water or 50.5 % w/w aqueous sucrose solution or the same aqueous sucrose solution containing 0.004 % w/w SDS. These mixtures were placed on a roller mixer overnight and the lower aqueous phase was sampled for fluorescence spectroscopy studies (excitation wavelength = 319 nm, scan speed = 240 nm min^–1^, PMT voltage = 950 V, excitation slit width = 5 nm and emission slit width = 5 nm).

### Determination of benzophenone concentration in 50.5 % w/w aqueous sucrose

0.1 M benzophenone was dissolved in 4.0 ml *n*-dodecane and hand-shaken with 4.0 ml of either pure water, 50.5 % w/w aqueous sucrose solution or the same aqueous sucrose solution containing 0.004 % w/w SDS. These mixtures were placed on a roller mixer for 2 h and the lower aqueous phase was sampled and diluted by a factor of two prior to UV spectroscopy analysis.

### Characterization

#### ^1^H and ^19^F NMR spectroscopy

^1^H and ^19^F NMR spectra were recorded in either (CD_3_)_2_CO, CDCl_3_ or CD_3_OD using a Bruker AV1-400 MHz spectrometer. Typically 64 scans were averaged per spectrum.

#### DMF GPC

Molecular weight distributions were determined using a DMF gel permeation chromatography (GPC) instrument operating at 60 °C that comprised two Polymer Laboratories PL gel 5 μm Mixed C columns and one PL polar gel 5 μm guard column connected in series to a Varian 390 LC multidetector suite (only the refractive index detector was utilized) and a Varian 290-LC pump injection module. The GPC eluent was HPLC grade DMF containing 10 mM LiBr and was filtered prior to use. The flow rate was 1.0 mL min^–1^ and DMSO was used as a flow-rate marker. Calibration was conducted using a series of 10 near-monodisperse poly(methyl methacrylate) standards (*M*_n_ = 625–618 000 g mol^–1^). Chromatograms were analyzed using Varian Cirrus GPC software.

#### THF GPC

Molecular weight distributions were determined using a THF GPC instrument operating at 30 °C that comprised two Polymer Laboratories PL gel 5 μm Mixed C columns, a LC20AD ramped isocratic pump and a WellChrom K-2301 refractive index detector operating at 950 ± 30 nm. The THF mobile phase contained 2.0 v/v% triethylamine and 0.05 w/v% 3,5-di-*tert*-4-butylhydroxytoluene (BHT) and the flow rate was fixed at 1.0 mL min^–1^ and toluene was used as a flow-rate marker. A series of ten near-monodisperse poly(methyl methacrylate) standards (*M*_n_ = 1280–330 000 g mol^–1^) were used for calibration. Chromatograms were analyzed using Varian Cirrus GPC software.

#### Dynamic light scattering

Dynamic light scattering (DLS) studies were performed using a Zetasizer Nano-ZS instrument (Malvern Instruments, UK) at 25 °C at a scattering angle of 173°. Copolymer dispersions were diluted in water, 65 % w/w glycerol/water mixtures or 50.5 % w/w sucrose/water mixtures prior to light scattering studies. The intensity-average diameter and polydispersity (PDI) of the diblock copolymer particles were calculated by cumulants analysis of the experimental correlation function using Dispersion Technology Software version 6.20. Data were averaged over ten runs each of thirty seconds duration.

#### Transmission electron microscopy

Transmission electron microscopy (TEM) studies were conducted using a FEI Tecnai G2 spirit instrument operating at 80 kV and equipped with a Gatan 1k CCD camera. Copper TEM grids were surface-coated in-house to yield a thin film of amorphous carbon. For samples prepared in *n*-dodecane the grids were then loaded with dilute copolymer dispersions (0.2 % w/w) and imaged without staining. For aqueous samples the grids were plasma glow-discharged for 20 seconds to create a hydrophilic surface prior to being loaded with dilute copolymer dispersion (0.2 % w/w). The sample-loaded grids were soaked in 0.75 % w/w uranyl formate solution (15 μl) for 20 seconds in order to improve contrast.

#### Laser diffraction

The volume-average droplet (D[4,3]) diameter was determined using a Malvern Mastersizer 2000 instrument equipped with a small volume Hydro 2000SM sample dispersion unit (*ca*. 100 mL), a He–Ne laser operating at 633 nm, and a solid-state blue laser operating at 466 nm. The stirring rate was adjusted to 1000 rpm in order to avoid creaming or sedimentation of the droplets during analysis. After each measurement, the cell was rinsed twice with isopropyl alcohol. The glass walls of the cell were carefully wiped to avoid cross contamination and the laser was aligned centrally to the detector prior to data acquisition.

#### Optical microscopy

Optical microscopy images were recorded using a Motic DMBA300 digital biological microscope equipped with a built-in camera and analyzed using Motic Images Plus 2.0 ML software.

#### Fluorescence microscopy

Fluorescence microscopy images were recorded on a Zeiss Axio Scope A1 microscope fitted with an AxioCam 1Cm1 monochrome camera using Zeiss filter set 43 HE (excitation 550/25 nm and emission 605/70 nm). Images were captured and processed using ZEN lite 2012 software.

#### UV-visible absorption spectroscopy

Visible spectra were recorded in transmittance mode between 800 and 400 nm for selected Pickering emulsions using a UV 1800 Shimadzu spectrophotometer. UV spectra were recorded using the same instrument.
