# Supplementary material for: Bespoke contrast-matched diblock copolymer nanoparticles enable the rational design of highly transparent Pickering double emulsions
Source: Nanoscale. 2016 Jul 6;8(30):14497–506. doi: 10.1039/c6nr03856e (PMC5047046; doi:10.1039/c6nr03856e)
Supplement: Supplementary file 1 [file NR-008-C6NR03856E-s001.pdf]

Electronic Supplementary Material (ESI) for Nanoscale.
This journal is © The Royal Society of Chemistry 2016

Supporting information for:

Bespoke contrast-matched diblock copolymer nanoparticles enable the rational design of highly transparent Pickering double emulsions

Matthew J. Rymaruk,[1*] Kate L. Thompson,[1] Matthew J. Derry,[1] Nicholas J. Warren[1], Liam P. D. Ratcliffe[1], Clive N. Williams[2], Steven L. Brown[2] and Steven P. Armes,[1*]

1. Dainton Building, Department of Chemistry, The University of Sheffield, Brook Hill, Sheffield, Yorkshire, S3 7HF, UK

2. *Scott Bader Company Ltd, Wollaston, Wellingborough, Northants, NN29 7RL, UK.*

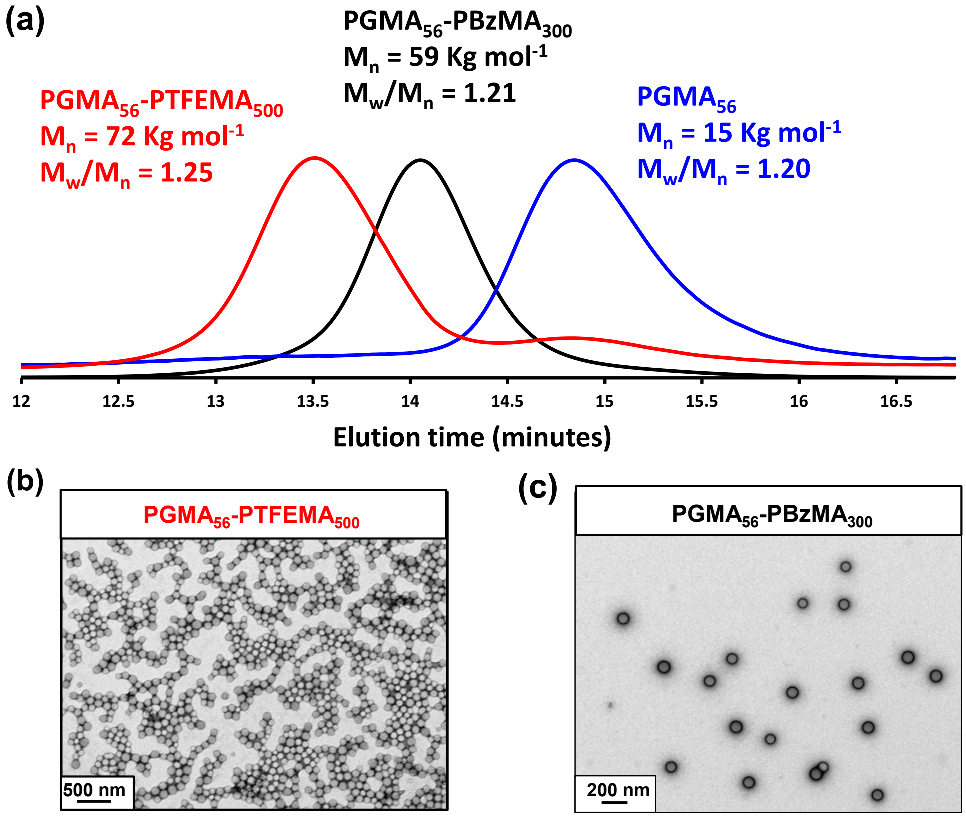

Figure S1. (a) DMF GPC chromatograms for the $PGMA_{55}$ macro-CTA used in this work, along with $PGMA_{56}$-$PTFEMA_{500}$ diblock copolymers and $PGMA_{56}$-$PBzMA_{300}$ diblock copolymers. (b) Representative TEM image of $PGMA_{56}$-$PTFEMA_{500}$ spherical nanoparticles. (c) Representative TEM image of $PGMA_{56}$-$PBzMA_{300}$ spherical nanoparticles

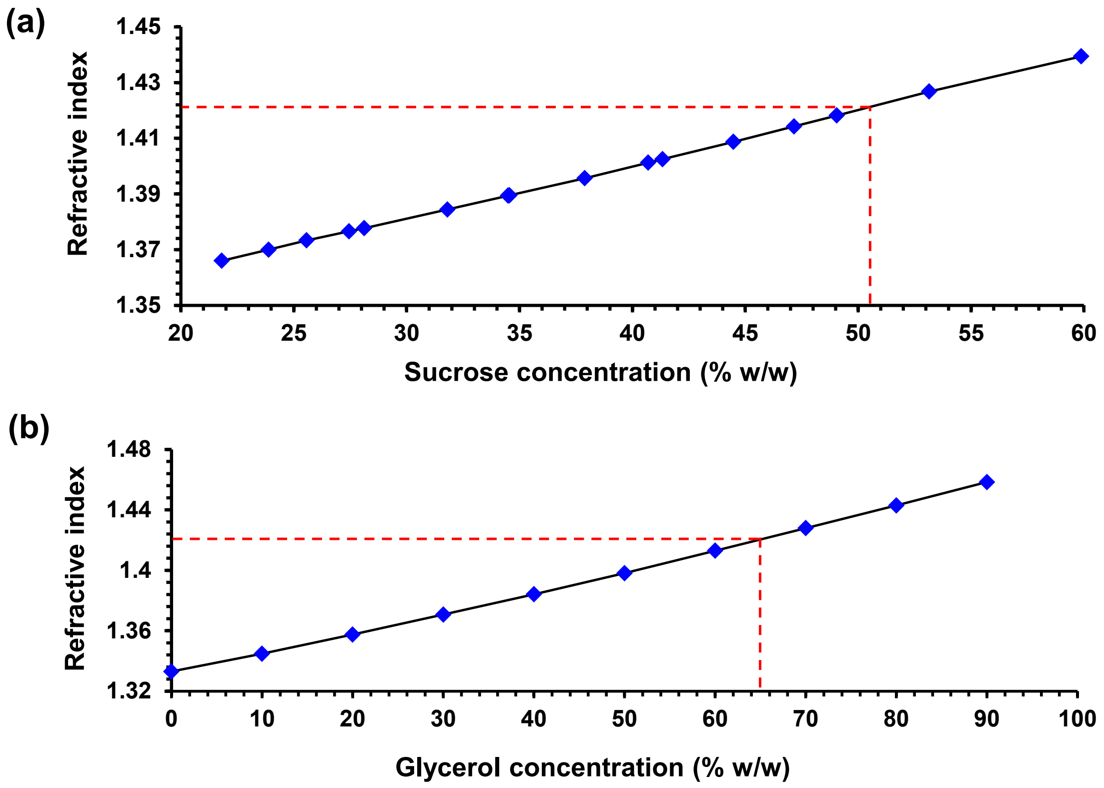

Figure S2. (a) Refractive index vs. sucrose concentration, obtained from the literature, for a range of aqueous sucrose concentrations.[1] The dashed red line indicates the refractive index of a 50.5 % w/w aqueous sucrose solution (i.e. contrast-matched with $PGMA_{56}$-$PTFEMA_{500}$) is 1.42. (b) Refractive index vs. glycerol concentration, obtained from the literature, for a range of aqueous glycerol concentrations.[2] The dashed red line indicates the refractive index of a 65 % w/w aqueous glycerol solution (i.e. contrast-matched with $PGMA_{56}$-$PTFEMA_{500}$) is 1.42.

(a)

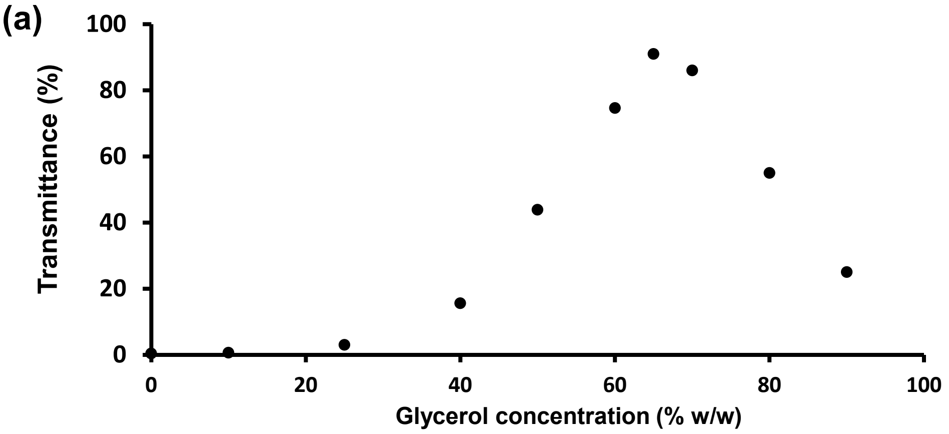

(b)

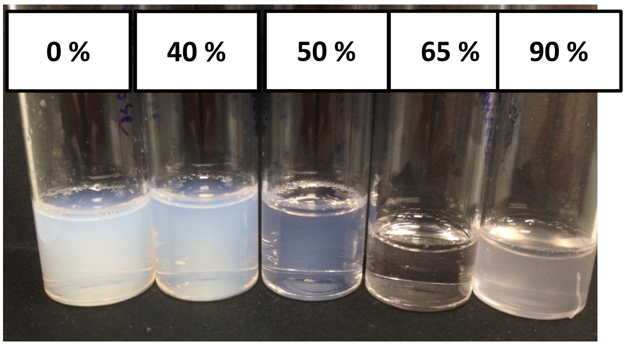

Figure S3. (a) Transmittance data obtained at 400 nm for a 2.0% w/w dispersion of $PGMA_{56}$-$PTFEMA_{500}$ nanoparticles as a function of glycerol concentration. (b) Corresponding digital images recorded for selected aqueous dispersions in the presence of various glycerol concentrations.

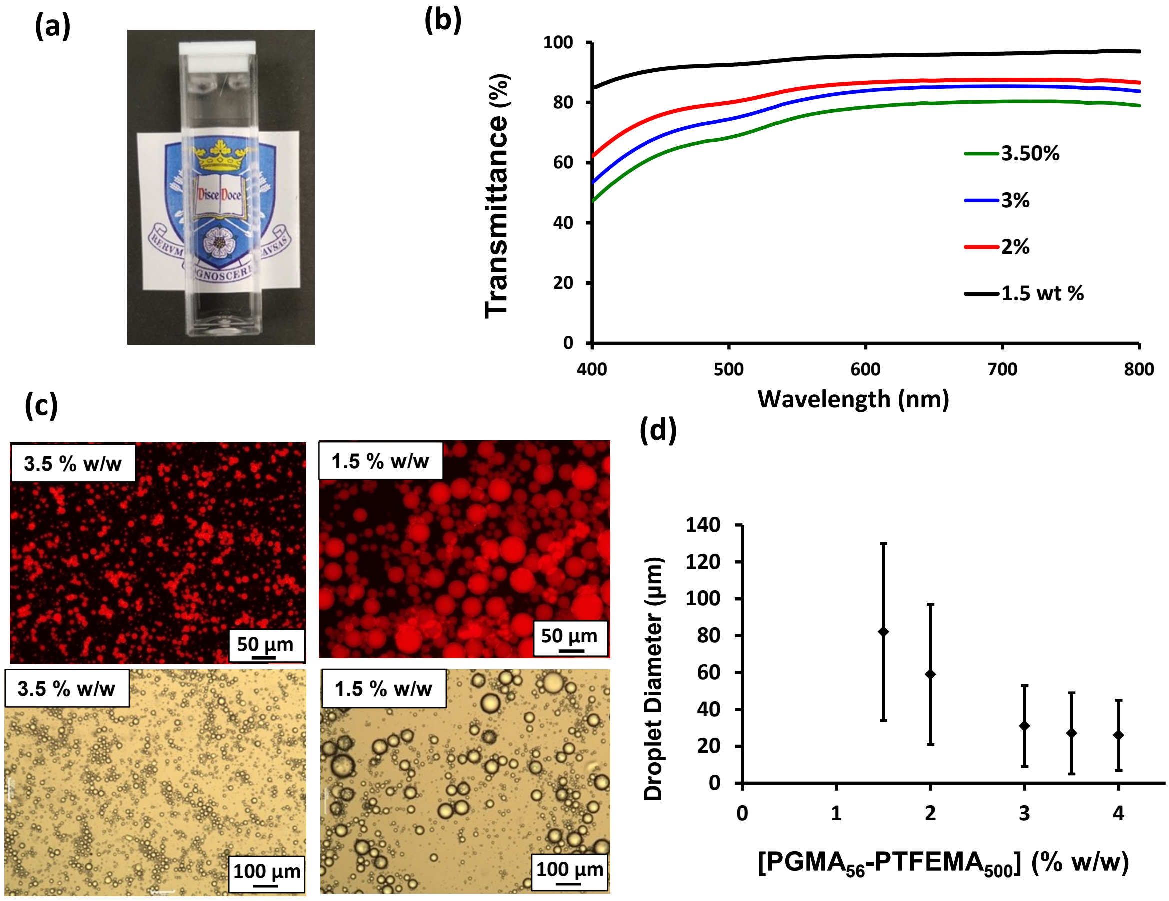

Figure S4. (a) Digital photograph of *n*-dodecane-in-aqueous glycerol (65 % w/w glycerol) Pickering emulsion prepared using 1.50 % w/w $PGMA_{56}$-$PTFEMA_{500}$ spherical nanoparticles. (b) Transmittance data obtained for a series of *n*-dodecane-in-aqueous glycerol (65 % w/w glycerol) Pickering emulsions prepared using 1.5 to 3.5 % w/w $PGMA_{56}$-$PTFEMA_{500}$ spherical nanoparticles. (c) Optical and fluorescence micrographs obtained for selected *n*-dodecane-in-65 % w/w aqueous glycerol (65 % w/w glycerol) Pickering emulsions prepared using either 1.5 or 3.5 % w/w $PGMA_{56}$-$PTFEMA_{500}$ spherical nanoparticles. For optical microscopy studies, emulsions were diluted using pure water to provide sufficient contrast for imaging. For fluorescence microscopy, Nile red was dissolved in the *n*-dodecane phase prior to high-shear homogenization. d) Variation in volume-average droplet diameter (as determined by laser diffraction) for a series of *n*-dodecane-in- aqueous glycerol (65 % w/w glycerol) Pickering emulsions as a function of $PGMA_{56}$-$PTFEMA_{500}$ copolymer concentration

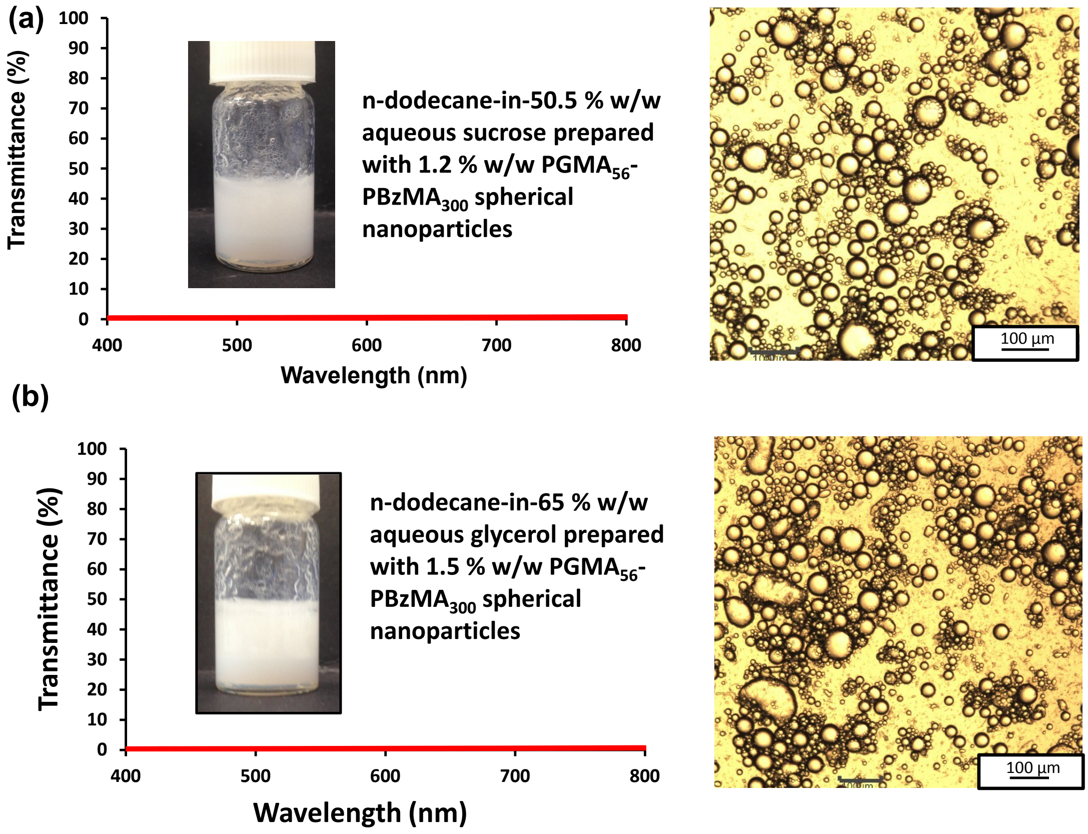

Figure S5. (a) Transmittance spectrum (left) and corresponding optical micrograph (right) recorded for an *n*-dodecane-in- aqueous sucrose (50.5% w/w sucrose) Pickering emulsion prepared using 1.20 % w/w $PGMA_{56}$-$PBzMA_{300}$ spherical nanoparticles (Inset: digital image depicting the turbid appearance of this control emulsion. (b) Transmittance spectrum (left) and corresponding optical micrograph (right) recorded for an *n*-dodecane-in- aqueous glycerol (65% w/w glycerol) Pickering emulsion prepared using 1.50 % w/w $PGMA_{56}$-$PBzMA_{300}$ spherical nanoparticles (Inset: digital image depicting the turbid appearance of this control emulsion. In both cases, the observed turbidity arises because PBzMA has a relatively high refractive index of 1.58, which is significantly greater than that of *n*-dodecane and either aqueous phase.

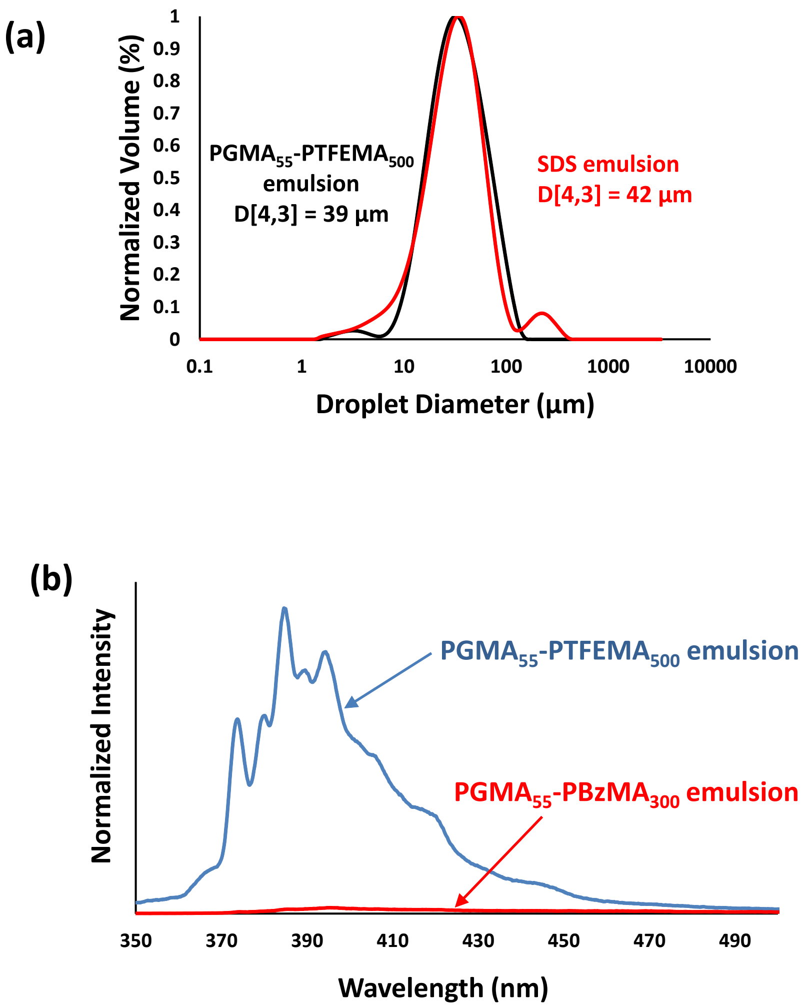

Figure S6. (a) Volume-average droplet size distributions obtained via laser diffraction for the contrast-matched Pickering emulsion (black curve) and surfactant-stabilized emulsion (red curve) used in the pyrene fluorescence experiments shown in Figure 5 of the main text. The similarity between these two size distributions confirms that surface area differences between these two emulsions are negligible. (b) Emission spectra recorded for two pyrene-loaded Pickering emulsions prepared using **isorefractive** $PGMA_{55}$-$PTFEMA_{500}$ nanoparticles dispersed in 50.5 % w/w aqueous sucrose(blue spectrum) and **non-isorefractive** $PGMA_{55}$-$PBzMA_{300}$ nanoparticles dispersed in pure water (red spectrum). The highly turbid nature of the latter dispersion leads to almost complete attenuation of the pyrene emission spectrum at a pyrene concentration of 20 µM.

(a)

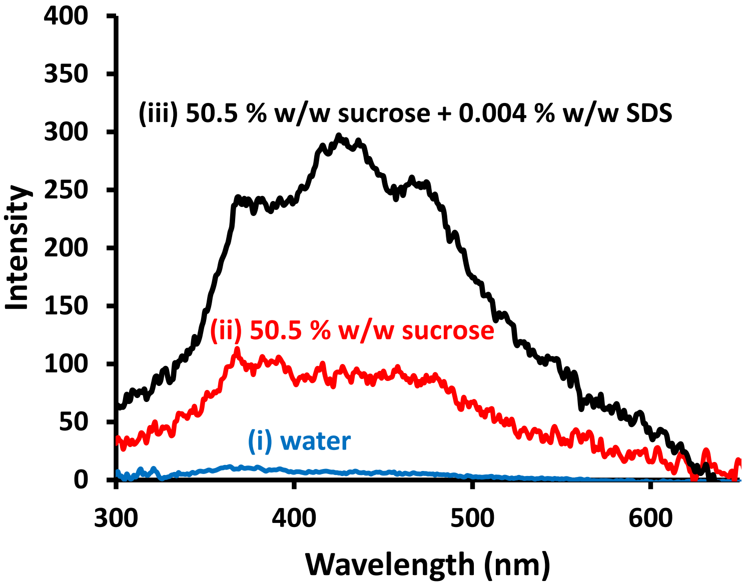

(b)

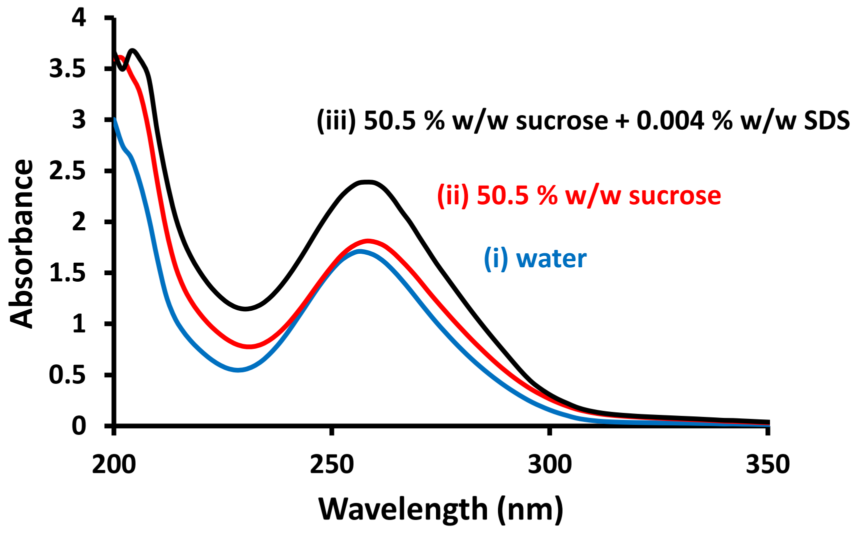

Figure S7. (a) Emission spectra recorded at 20 °C for: (i) a pyrene-saturated aqueous solution (intensity at 384 nm = 7 units); (ii) a pyrene-saturated 50.5 % w/w aqueous sucrose solution (intensity at 384 nm = 80 units); (iii) a pyrene-saturated 50.5 % w/w aqueous sucrose solution containing 0.004 % w/w SDS (intensity at 384 nm = 287 units). (b) UV spectra recorded at 20 °C for: (i) a benzophenone-saturated aqueous solution (absorbance at 256 nm = 1.71); (ii) a benzophenone-saturated 50.5 % w/w aqueous sucrose solution (absorbance at 256 nm = 1.79); (iii) a benzophenone-saturated 50.5 % w/w aqueous sucrose solution containing 0.004 % w/w SDS (absorbance at 256 nm = 2.37).

References:

1 D. F. Charles, *Anal. Chem.*, 1965, **37**, 405–406.

2 L. F. Hoyt, *Ind. Eng. Chem.*, 1934, **26**, 329–332.
